# The development of high school students’ statistical literacy across grade level

**DOI:** 10.1007/s13394-023-00449-x

**Published:** 2023-04-17

**Authors:** Achmad Badrun Kurnia, Tom Lowrie, Sitti Maesuri Patahuddin

**Affiliations:** STEM Education Research Centre (SERC), Faculty of Education, University of Canberra, Bruce, Canberra, ACT 2617 Australia

**Keywords:** Statistical literacy, Assessment, High school students, Cross-sectional study, Gender

## Abstract

The capacity to interrogate data with critical thinking is a strong predictor of statistical literacy (SL). This data interrogation, from the data consumers’ perspective, incorporates four complex response skills: *interpreting*, *communicating*, *evaluating*, and *decision-making*, and those skills are strongly supported by students’ appreciation of three interrelated knowledge components (*text and context*, *representation*, and *statistical-mathematical knowledge*). Due to the need to be critical data-information readers, students’ SL should develop during their formal schooling. The aim of this paper was to investigate differences in SL between Indonesian year 9 and year 12 students and between female and male students. The same test was administered to 48 year 9 students (50% females) and 48 year 12 students (50% females) from 16 different schools in Indonesia. Findings revealed that the highest percentage of year 9 and 12 students demonstrated evidence of *consistent but non-critical* thinking (level 4), suggesting that they exhibited their statistical knowledge but not in critical ways. There were 42% of year 9 students showing limited statistical thinking (levels 1 to 3) compared to 17% of year 12 students. Furthermore, while there were no significant gender differences in students’ SL and its all skills, the study shows significant grade level differences in overall SL as well as in its skills except *interpreting*. Implications of this study include the development of a framework that provides a coherent assessment of students’ SL from a data consumers’ perspective, along with suggestions for classroom teaching.

The advances in technology that allow for a rapid spread of data-based information require students to be statistically literate. Being statistically literate means being able to critically respond to information involving statistics, which can be challenging for students (Shields, 2005). Increasingly, students are being bombarded with data information, heightened during the COVID-19 pandemic (e.g., da Silva et al., 2021; Watson & Callingham, 2020). Almost every day they are presented with predictions and claims which are based on the number of COVID-19 cases, deaths and recovered, and the vaccination program. Furthermore, as students move into the workplace, understanding and interpreting statistics is required (Moreno, 2002). Hence, a solid level of statistical literacy (SL) is becoming increasingly important for high school students.

Students’ capacity to interrogate data with critical thinking is essential for well-informed citizens (e.g., Gal, 2002; Organisation for Economic Cooperation and Development [OECD], 2014). However, there has been a clear trend suggesting that the majority of high school students from most developing countries perform poorly in problems involving statistics (OECD, 2004, 2014). Such trends have been revealed in Programme for International Student Assessment (PISA) reports on the *uncertainty and data* subscale over the past 2 decades. In the PISA 2003 report, the six countries in the lowest rank were all developing countries in which 50 to 80% of their students were in level one and below level one out of PISA’s six levels on this subscale (OECD, 2004). Level one of PISA on this subscale suggests that students are able to locate specific data values from a simple representation, while below level one is an additional level to accommodate students who could not achieve level one.

One decade later, in the PISA 2012 test, the students in those countries did not show any significant progress on the *uncertainty and data* subscale (OECD, 2014). For example, Indonesia was ranked 38th of 40 participating countries with around 72% students in level one and below level one in the PISA 2003 test; while in the PISA 2012 test, Indonesian students (aged 15–16 years old, majority in year 9) were the 63rd rank of 64 participating countries, with around 73% of them performing in level one and below level one. Moreover, no gender difference was found in the Indonesian students’ performances over such a period of time, meaning both females and males performed poorly in the *uncertainty and data* subscale. It is concerning that the majority of students from Indonesia and other developing nations remain near the bottom of the rankings in the *uncertainty and data* comprehension, despite the importance of these skills in an increasingly data-driven world.

The underperformance of students aged 15–16 years old from developing countries has raised another concern on whether the students in the latter years of formal schooling show sufficient progress in solving data-based problems. Given that all students are expected to leave school as statistically literate citizens (Gal, 2002; Watson & Callingham, 2020), it is critical to understand the SL of final year students. However, data on developing countries rarely reported students’ SL in the last years of schooling. Studies on statistics education involving upper high school or final year students have been mainly conducted in western contexts (e.g., Budgett & Rose, 2017; Dierdorp et al., 2017; Gil & Gibbs, 2017) with insufficient studies having been conducted in non-western contexts (e.g., Aoyama, 2007; Hafiyusholeh et al., 2018; Sharma, 2014). Therefore, further studies need to be conducted to assess the SL of upper high school students from non-western and non-developed countries due to their different characteristics and cultures.

Multiple assessment frameworks have been established, and assessment studies have been conducted in order to assess the SL skills of high school students (e.g., Aoyama & Stephens, 2003; Callingham & Watson, 2017; Mooney, 2002; Mullis et al., 2012; Pfannkuch, 2005; Yolcu, 2014). Several studies, for instance, have investigated the level of SL attained by students in the same grade (e.g., Mullis et al., 2012; Pfannkuch, 2005), while some others provide the levels achieved by students from different grade levels (e.g., Aoyama & Stephens, 2003; Callingham & Watson, 2017; Yolcu, 2014). Of those involving students from different grades, Aoyama and Stephens (2003) conducted a study with years 5 and 8 students and claimed that the improvement across years 5 and 8 is unlikely to be attributed to formal statistical education due to the lack of statistical treatment between the two grades; instead, it might be attributed to cognitive development in general including students’ experiences with data-based information in and out of class. Callingham and Watson (2017) conducted a longitudinal study of children in years 5 to 10 and discovered that there was very limited growth from years 5 to 6 and years 9 to 10; however, there was growth (although minor) throughout the transition from primary to secondary school (years 6 to 7). Finally, Yolcu (2014) found no significant grade level differences across years 6 to 8, and this lack of differences between grades might be caused by the spiral curriculum in middle school mathematics. All those studies were conducted in developed countries and did not involve students from the final year of schooling.

The present cross-sectional study was conducted to investigate the critical responses exhibited by Indonesian year 9 and year 12 students. The year 9 and year 12 students were chosen as they represent Indonesian students participating in the PISA test and final year of schooling, respectively. Moreover, the present study also intended to understand whether the Indonesian students’ SL is influenced by their gender.

## Gender differences in students’ statistical literacy

Although many studies have been conducted to investigate school students’ SL, few studies were conducted on the effect of gender on the students’ SL. Moreover, some studies on gender differences focused on the students’ interest in or attitudes towards statistics (e.g., Carmichael & Hay, 2009; Chiesi & Primi, 2015) rather than on the students’ SL levels. Few studies have investigated the effect of gender on the students’ SL levels. For instance, Watson and Moritz (2000) conducted a study in Australia with students in years 3–11, while Yolcu (2014) conducted a study in Turkey for students in years 6–8. In addition, both PISA 2003 and 2012 provided a broader picture of gender differences on the *uncertainty and data* subscale (OECD, 2004, 2014). The PISA reports covered the students’ levels for the *uncertainty and data* subscale among the participating countries based on gender. Those studies or reports enabled further investigation of the trends that occurred over a decade as elaborated below.

The findings from the previous studies on the effect of gender on students’ SL partly showed consistency. In the *uncertainty* subscale of PISA 2003, gender differences were visible for 24 out of the 30 OECD countries; in addition, it was revealed that males outperformed females in most countries (OECD, 2004). Australia and Indonesia were among the countries with no gender differences. In PISA 2012, the general trend was males continued to outperform females on the *uncertainty and data* subscale across the participating countries (OECD, 2014). However, the trend for Indonesia remained unchanged (i.e., no gender differences). Furthermore, it is important to note that these trends are not consistent across all studies. For example, Watson and Moritz (2000) and Yolcu (2014) found performance differences in favor of females in Australia and Turkey, respectively, in studies that examined SL knowledge. These conflicting results highlight the fact that different SL tasks and the context that surround those tasks can produce different patterns in students’ performances.

As well as investigating the development of SL in Indonesian years 9 and 12 students, the present study also intended to investigate further evidence on gender differences in SL. Particularly, this study examined whether the students’ SL differs by non-adjacent grades and by gender. The involvement of years 9 and 12 students was expected to portray the differences, and this is in line with Yolcu’s (2014) recommendation to not involve students from the adjacent grades when observing the development of students’ SL. The involvement of students from the adjacent grades might result in failure to demonstrate the development being investigated due to insufficient duration to confidently assess change.

Performance differences in relation to gender have been a focus of international mathematics test such as PISA (including the *uncertainty and data* subscale). However, specific studies investigating gender differences on SL are rather scarce (Yolcu, 2014), especially in developing countries. Although gender differences were not found in the Indonesian students’ performance in the *uncertainty and data* subscale for the past 2 decades (from PISA 2003 to PISA 2012), Indonesian female and male students underperformed in this subscale. Moreover, these findings are outdated. Consequently, it was deemed appropriate to investigate the gender variable in order to provide a current perspective on this variable.

## Frameworks for assessing students’ statistical literacy

The reviews on the existing efforts to assess students’ SL resulted in two major perspectives from the six existing frameworks as summarized in Table 1, namely, data producers and data consumers. The data producers’ perspective focuses on assessing students to think as “young” statisticians to solve statistical problems (Franklin et al., 2005). In contrast, the data consumers’ perspective examines students’ ability or skill to respond to statistical information. The responses reveal each student’s ability to, such as, understand and evaluate statistical information (Wallman, 1993), make personal daily choices based on the news on media (Franklin et al., 2005), critically evaluate relevant news on media (Budgett & Rose, 2017; Guler et al., 2016), and interpret, critically evaluate, and communicate statistical results from diverse sources (Gal, 2002). As observed in Table 1, the frameworks that take the data producers’ perspective have a common hierarchical structure for assessing performance, namely, a three-step hierarchical categorization. By contrast, the categorization of hierarchical levels that take data consumers’ perspective is varied. The frameworks in this perspective range from four to six categorization levels.

**Table 1:** The six existing SL frameworks

The framework	SL perspective	The construct	The hierarchical levels
The Guidelines for Assessment and Instruction in Statistics Education (GAISE) (Franklin et al., 2005)	Data producers	SL is elaborated into four statistical problem-solving processes: *formulate questions*, *collect data*, *analyze data*,* and interpret results*	Levels A, B, and C
The Levels of Conceptual Understanding in Statistics (LOCUS) (Whitaker et al., 2015)	Data producers	*Same with GAISE*	Levels A, B, and C
Mooney’s Framework (Mooney, 2002)	Data consumers	SL is elaborated into four constructs: *describing*, *organizing and reducing*, *representing*, and *analyzing and interpreting data*	Idiosyncratic, transitional, quantitative, and analytical
Watson and Callingham’s Framework (Watson & Callingham, 2003)	Data consumers	Tier 1 (understanding basic statistical term), Tier 2 (understanding basic statistical concept in context), Tier 3 (using critical thinking)	Idiosyncratic, informal, inconsistent, consistent non-critical, critical, and critical mathematical
Trends in International Mathematics and Science Study (TIMSS) (Mullis et al., 2012)	Data consumers	Involved three students’ cognitive domains: *knowing*, *applying*, and *reasoning*	Low, intermediate, high, and advanced
Programme for International Student Assessment (PISA) (OECD, 2014)	Data consumers	Included three process categories: *formulate*, *employ*, and *interpret/evaluate*	Levels 1 to 6

**Fig. 1:**
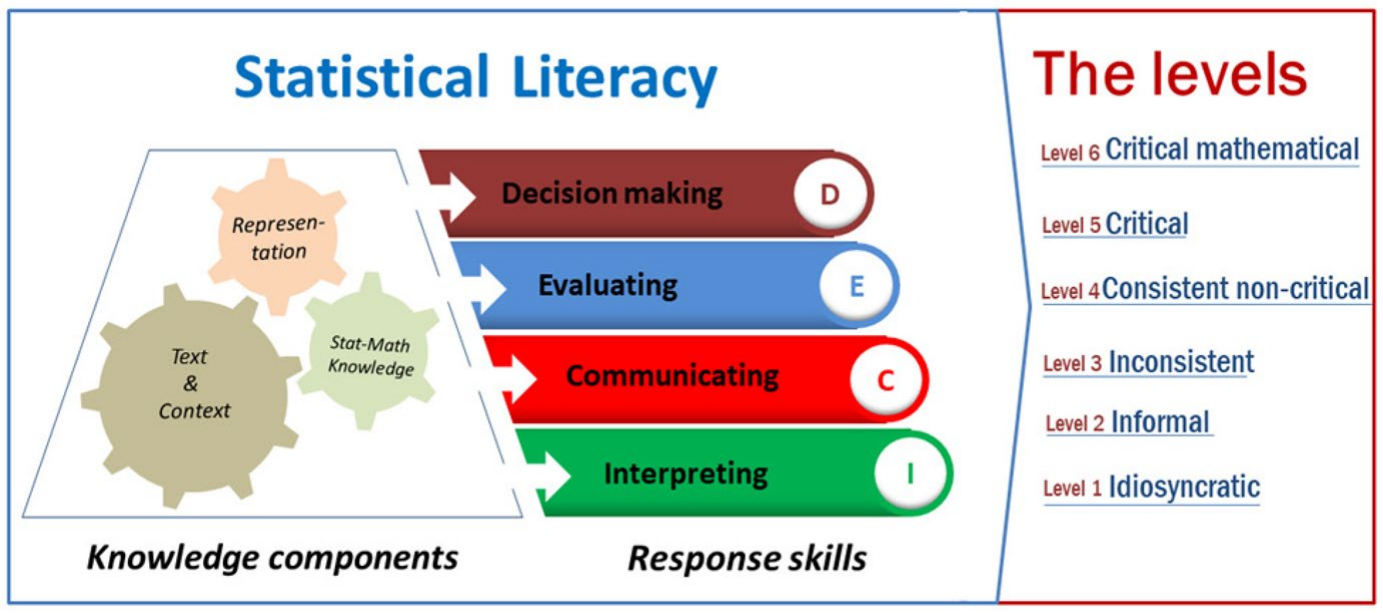
The theoretical framework of statistical literacy

Informed by the previous frameworks, we developed an SL framework (see Fig. 1) to guide instrument development and data analysis for our investigation of students’ SL. This study’s conceptual underpinnings were based on the data consumers’ perspective as the majority of students engage often with data-driven information. With regard to the constructs, research indicates that being critical consumers of quantitative information involves four response skills that determine students’ SL. These four skills are data interpretation, data communication, data-based evaluation, and data-driven decision-making (e.g., Budgett & Rose, 2017; Franklin et al., 2005; Gal, 2002; Guler et al., 2016; Wallman, 1993). Some researchers have further emphasized that students’ SL is greatly influenced by their understanding of the three knowledge components (*text and context, representation*, and *statistical-mathematical knowledge*) (Gal, 2002; Watson, 2006).

The SL skills aim to assess different type of students’ responses to information containing statistics. The skill of *interpreting* statistical information involves an in-depth comprehension of the meaning of the data (Rumsey, 2002). It is in part shown by the ability to extract qualitative meaning from data that are frequently presented quantitatively (Aoyama & Stephens, 2003). *Communicating* the omnipresent data-based information involves sharing or discussing an understanding of the quantitative information with others so that they get properly informed (Gal, 2002). However, communication needs to be conducted effectively to help others understand the information appropriately (Krishnan, 2015). In certain contexts, students are provided with statistical claims or arguments to reason with. In this situation, critically *evaluating* statistical information is required to either support or refute such arguments or claims (Brown et al., 2010). Finally, *decision-making* is required on a daily basis by all data consumers including students when making, for example, personal choices (Krishnan, 2015).

Furthermore, the students’ appreciation of three knowledge components (*text and context*, *representation*, and *statistical-mathematical knowledge*) could support them in possessing the four complex response skills. Those three components do not support students’ response as separate entities, rather they are interconnected. Students’ appreciation of the *text and context* relates to their ability to navigate through texts in an attempt to comprehend the underlying context (Gal, 2002). Students’ appreciation of the representation of statistical information such as graphs or tables proved to be an important component contributing to students’ SL as those two representations are ubiquitous (Aoyama & Stephens, 2003) and often convey rich and dense information. Finally, having a sufficient level on some statistical concepts and the mathematical procedures enable students to have a correct interpretation of numbers used in a statistical report (Gal, 2002).

Considering the working perspective (data consumers) and constructs (four response skills and three components) of SL in this study, the most appropriate leveling system is that identified by Callingham and Watson (2017) and Watson and Callingham (2003). A hierarchy of six levels was established: from *idiosyncratic*, *informal*, *inconsistent*, to *consistent non-critical*, *critical*, and *critical mathematical*. This hierarchy describes the progress of students’ SL from the perspective of data consumers and more importantly reflects the students’ progress on the three components of SL: *text and context*, *representation*, and *statistical-mathematical knowledge*. Based on Watson (2006), at the idiosyncratic level, students show personal engagement with context and one-to-one counting and reading values; at the informal level, students show colloquial engagement with context and basic one-step calculation using data from the table and graph; while at the inconsistent level, students show selective engagement with context and are likely to apply content knowledge inappropriately or without statistical reasoning. These three first levels indicate students’ limited knowledge on the three components. Furthermore, at the consistent non-critical level, students show appropriate responses but without critical engagement with context and a reasonable application of statistical and mathematical concepts. At the critical level and critical mathematical level, students show the ability to produce critical responses. The difference between these two levels is the complexity of the reasoning. Detailed descriptions of the six levels of SL are presented in Appendix A.

In conclusion, the purpose of this framework was to examine the extent to which the SL proficiency levels of Indonesian students vary by grade and gender. Specific research questions guiding this investigation are the following: (1) What levels of SL do Indonesian high school students possess? (2) Is there a significant difference in SL and skill level between female and male students? (3) Is there a significant difference in SL and skill level between year 9 and year 12 students?

## Method

### Participants

Participants were recruited in June 2019 consisting of a sample of 48 year 9 students (50% females) and 48 year 12 students (50% females) from 16 different schools. Table 2 shows the distribution of the 96 students selected using convenience and stratified purposive sampling (Onwuegbuzie & Collins, 2007; Robinson, 2014; Suri, 2011). Both sampling methods enabled the researcher to recruit participants from the accessible schools while ensuring the participants’ heterogeneity. The 16 schools were originated from two cities in East Java province of Indonesia (i.e., Surabaya and Jombang). Surabaya is the capital city of East Java which represents a metropolitan city, while Jombang represents a non-metropolitan city. In addition, the schools also represented schools under two ministries (the Ministry of National Education [MoNE] and the Ministry of Religious Affairs [MoRA]) and two school status (state and private schools). In summary, the participants were distributed into 50% from Surabaya, 50% under MoNE, and 50% state school. The six participants from each school were selected to represent three levels of knowledge (low, medium, and high) and the same number of female and male students by the help of their teachers. An ethical approval for this study was obtained from the University of Canberra Human Research Ethics Committee (Registration number: 1576).

**Table 2:** The distribution of participants

	Jombang				Surabaya			
	*MoRA*		*MoNE*		*MoRA*		*MoNE*	
	State	Private	State	Private	State	Private	State	Private
Year 9	6	6	6	6	6	6	6	6
Year 12	6	6	6	6	6	6	6	6

### Research instruments and data collection

An instrument was developed to measure (1) three knowledge components (*text-context*, *representations*, and *statistical-mathematical knowledge*) and (2) four response skills (*interpreting*, *communicating*, *evaluating*, and *decision-making*). The items were adapted from various resources following the stages as illustrated in Table 3. According to Ralston et al. (2018), a careful and systematic process in developing the assessment starts from a meticulous description of the construct to be assessed. Therefore, the SL framework was initially provided in this study to guide the instrument development.

**Table 3:** The item development phases

Stage	Description	Output
Generation	Analyze the Indonesian mathematics curriculum, mathematics textbooks, and Indonesian national examination for mathematics	Curriculum goals, basic skills for statistics, typical statistical tasks, and statistical topics to be included
Selection	Analyze some potential items from different sources	14 selected items
Adjustment	Adapt and adopt the selected items	The adapted 14 items
Pilot interview I	Examine the clarity of the items and the cognitive processes of four students with average academic achievement	The revised 14 items
Pilot test	Trial the items with twelve (low- to high-achieving) students to determine test administration process, test duration, and to understand students' written responses	Test administration procedure, test duration, and sample of students’ written responses
Pilot interview II	Investigate the cognitive processes underlying the written responses of students on a pilot test to enhance the quality of an interview protocol	10 revised items with their interview protocol

The first stage undertaken in developing items followed a method by Van den Heuvel-Panhuizen (1996). The method consists of three major steps: *generation*, *selection*, and *adjustment* (as presented in Table 3). The process of *generation* was employed to select the statistics topics to be included in the test. The second stage, *selection*, requires item selection from various resources by considering its appropriateness to the framework. All those items were in the form of statistics information involving reports, claims, arguments, or opinions. Finally, in the *adjustment* stage, those selected items were modified before being piloted.

After the initial version of items was finalized, a series of piloting was conducted as presented in Table 3. Pilot interview I involved four students, while pilot test and pilot interview II involved 12 students. The piloting aimed to provide evidence on whether the test items were clear for the respondents (Tiruneh et al., 2017). In addition, interviewing is also a method used to examine if the respondents comprehend and respond to items as intended by the researcher (Willis, 2005). In summary, the pilot helped the researcher to examine whether the students performed one of the four skills (*interpret*, *communicate*, *evaluate*, and *make decision*) when they were asked to do so. The piloting resulted in a final version of the ten items.

Finally, the test was administered in the first semester of 2019 (from July to October). The test was conducted in the participants’ own school and administered and supervised by the first author. This test was set in 120 min, but many participants managed to finish in their first 90 min. Among the ten items, four items were associated with interpreting (I), two items with communicating (C), two items with evaluating (E), and two items with decision-making (D). Table 4 exemplified the item for each response skill with the label I, C, E, or D, respectively.

**Table 4:**
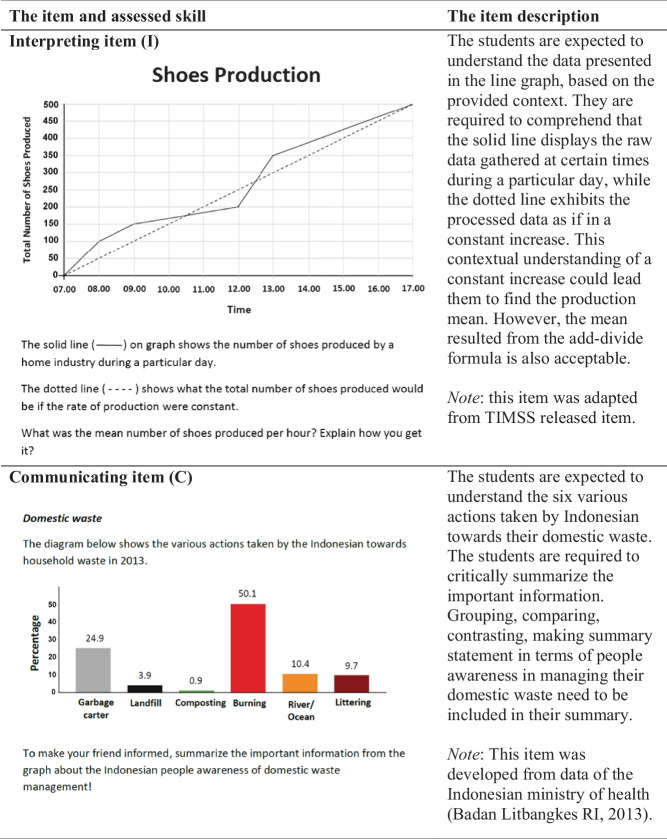

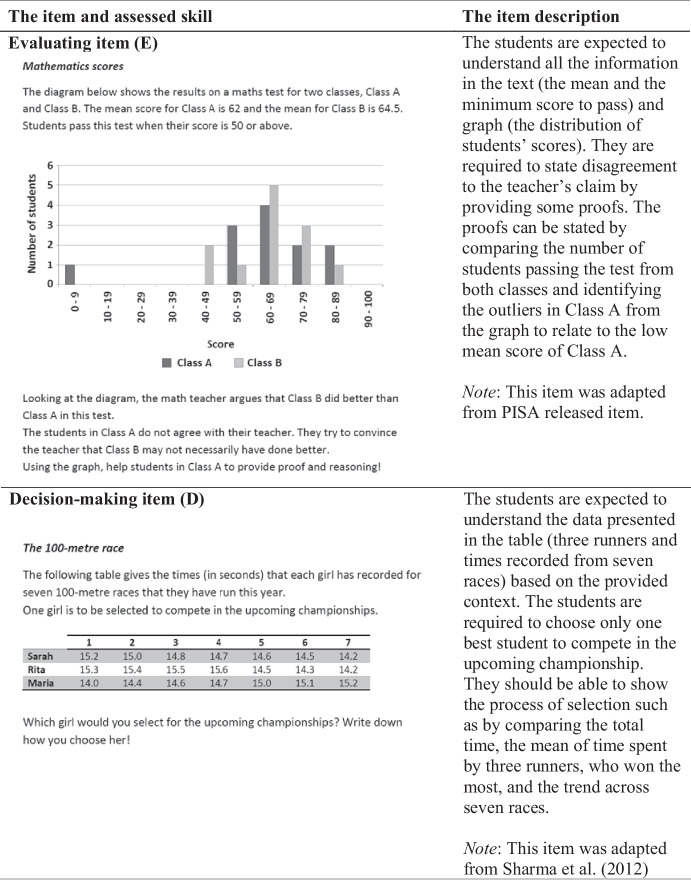
The exemplified items based on four skills

### Data analysis

As previously described, the data collections involved 96 students solving ten SL tasks. However, only eight items were analyzed, resulting in 768 unit analyses. Two out of four items assessing *interpreting* skill were excluded from the analysis. This exclusion considered that the other skills (*communicating*, *evaluating*, and *decision-making*) were also represented by only two items. Moreover, it considered only one representation for each skill: a line graph for interpreting, a bar graph for communicating, a bar graph for evaluating, and a table for decision-making. A series of data analysis procedures were performed on the data corpus. The double coding principle (adapted from Jones et al., 2000; Miles & Hubberman, 1994; Mooney, 2002) was applied to the students’ written responses by three coders (the first author and two trained coders). Three stages of coding were undertaken as explained below followed by how the results would be presented.

#### Group coding

In the group coding, the three coders encoded the written responses of 25% of the respondents. These respondents represented students with different levels of knowledge as well as a variety of responses. This coding process was guided by the assessment rubric (see the example in Appendix A, and this rubric was developed deductively during the literature review and justified through expert validations and limited trial. The group coding process began by assigning code to each of the three components contributing to the students’ SL (*text and context*, *representation*, and *statistical-mathematical knowledge*). The numerical code to be attached represents the level ranging from L1 (idiosyncratic), to L2 (informal), L3 (inconsistent), to L4 (consistent non-critical), L5 (critical), and L6 (critical mathematical).

#### Independent coding

Having practiced coding written responses for 25% of the students, the three coders continued coding the responses of the remaining 75% of students independently. Each of the three coders applied the same coding techniques as employed during the group coding stage. If the rubric still did not apply to particular students’ responses, such responses were re-examined thoroughly until the closest corresponding descriptor could be identified. Otherwise, a record was made by the coder individually to be discussed in the consensus coding (Stage 3).

Following up the individual coding, inter-rater analyses were conducted to check how strong the agreement was between the three coders. Kendall’s W was run to determine if there was an agreement between the three coders on the codes they provided to each of the three components. Kendall’s W (Gearhart et al., 2013; Laerd Statistics, 2016) was chosen because there were 3 coders, 72 students, and 3 variables (the three components contributing to students’ SL). The results showed that the three coders statistically significantly agreed in the codes they provided,* p* < 0.0005, and the resulted Kendall’s W coefficient was ≥ 0.814 with most values above 0.90 which were considered to be very strong agreement.

#### Consensus coding

As the three coders did not reach 100% agreement on the individual coding stage, the disputed codes were discussed in the consensus coding. Each consensus coding always started from listening to the reasoning of the coder coding differently. After the hearing session, the other two coders gave responses and the discussion followed for consensus. In the case of complete disagreement, any coder could voluntarily start to present their reasoning followed up by a consensus discussion.

The median of the three components’ codes would have further characterized the code (or level) for each item, each skill, and overall SL. The median was chosen instead of the mean as it is the recommended measure of central tendency for ordinal data (Boone & Boone, 2012; Harpe, 2015; Joshi et al., 2015; Stevens, 1946). In case the median is halfway between the two levels, it was rounded down to ensure that participants’ responses were coded to the nearest corresponding descriptors. This rounding followed Mooney (2002) who rounded down the mean which is halfway between two levels to determine the students’ statistical thinking level from various constructs. For example, if a participant’s *text and context* knowledge on two *interpreting* items were coded as 4 and 4, *representation* as 4 and 5, and *statistical-mathematical* knowledge as 5 and 5, its median would be 4.5 = [(4 + 5) ÷ 2]. The median would then be rounded down to the lower level, resulting in the participant receiving a level 4 on the interpreting skill (i.e., inconsistent). The same process as mentioned above was further applied to find the other skills’ code and the student’s SL code (level). Finally, the participant’s overall SL level has been derived from the median of all codes they obtained in all items.

#### Presenting the results

In reporting the students’ SL levels, the distribution of levels achieved by the students was presented using a table. In that table, the six levels were classified into two groups: the lower group that consists of L1 (idiosyncratic) to L3 (inconsistent) and the upper group that consists of L4 (consistent non-critical) to L6 (critical mathematical). The two major classifications (i.e., lower and upper group) were inspired by the four levels of students’ SL of Sharma et al. (2012) who merged the first three levels of Watson and Callingham into one to illustrate the students’ low performances. In addition, the top three levels were used by researchers to measure the expected SL of people around the world (Klein et al., 2016; Tarran, 2017). In this study, those top three levels (the upper group) reflect the students’ appropriate responses to the data-based information. Subsequently, a series of Mann–Whitney *U* test was performed following guidance from Laerd Statistics (2015) to investigate whether there was a difference in the students’ SL levels based on gender and grade level since the data were not normally distributed (Hollander et al., 2013). In this analysis, the data violated one assumption (i.e., the distributions of the two groups of the independent variable were not similarly shaped), therefore the mean rank was analyzed to determine whether there were any statistically gender and grade level differences in the students’ SL level.

## Results

### Students’ statistical literacy levels

Table 5 displays the distribution of the students’ SL levels across the hierarchy segregated by grade levels. Furthermore, such distribution of students’ SL was presented into two groups: the lower group (L1 to L3) showing the students’ responses with limited statistical thinking and the upper group (L4 to L6) showing the students’ responses with statistical thinking (see the example of students’ written works in Appendix B). Students were more likely to demonstrate non-critical thinking (L4) than critical thinking (L5) in both year 9 and year 12. In addition, the spread of students’ SL levels was limited to five levels (year 9) and four levels (year 12).

**Table 5:** The distribution of year 9 and year 12 students’ SL level

Year	Lower group			Upper group		
	L1 N (%)	L2 N (%)	L3 N (%)	L4 N (%)	L5 N (%)	L6 N (%)
Y-9 Y-12	1 (2%)	2 (4%)	17 (36%)	26 (54%)	2 (4%)	0 (0%)
	0 (0%)	1 (2%)	7 (15%)	32 (66%)	8 (17%)	0 (0%)

The number of year 9 and year 12 students who performed in the lower and upper groups was proportionally different. With respect to year 9 students, the lower and upper groups were comparable with approximately a half of year 9 students (42%) involved in limited statistical thinking (L1, L2, and L3) when responding to data-based information. In contrast, the number of year 12 students in the upper group was approximately five times those in the lower group. This indicates that the majority of year 12 students (83%) could appropriately involve statistical thinking when responding to data-based information, while a smaller proportion (17%) demonstrated limited statistical thinking.

Proportional differences were also observed across grade levels. The number of year 9 students in the L3 (inconsistent) was twice more than those of the year 12. In the upper group, both the year 9 and year 12 students could already achieve L4 and L5. However, year 12 students showed a higher percentage than year 9 students in both L4 (consistent non-critical) and L5 (critical). From this comparison, it thus confirmed that the higher-grade students (year 12) performed better in SL levels.

Noteworthy, although the year 12 students indicated a better performance than the year 9 students, the patterns in both grade levels were almost similar. First, there were no students in both grades who could achieve the highest level (critical mathematical). Second, the highest proportions in each grade level were in the L4 (consistent non-critical) which was achieved by 54% of year 9 students and 66% of year 12 students.

### Students’ level in the four response skills

**Fig. 2:**
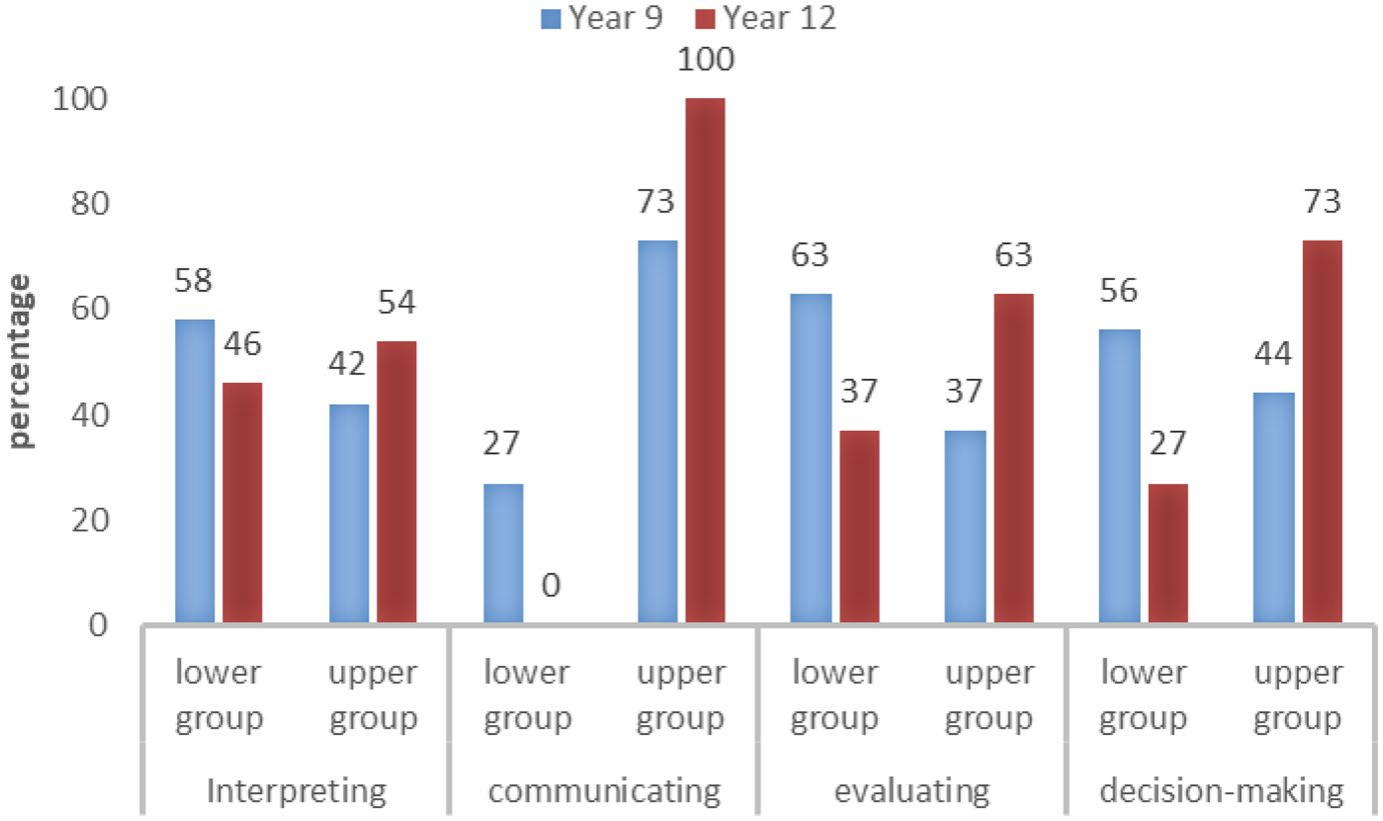
The percentage of students by grade level and skill

Figure 2 represents the proportion of students who were performing in both lower and upper groups by grade level and skill. Two conclusions can be derived, namely, (1) year 12 students performed better than year 9 students in all skills and (2) with the *communicating* skill the most frequently demonstrated. The following paragraphs briefly justify and discuss these conclusions.

The graph highlights that there were more year 12 students than year 9 students in the upper levels for skills acquisition by SL. It also reveals that students from both grade levels showed better performance in *communicating* skill than the other three skills. This suggests that students in both grades engaged with the *communication* skill in a more sophisticated manner than the other skills. Table 6 further provides evidence that the majority of year 9 and year 12 students performed L4 in *communication* skill. This suggests that the majority of students were able to make sense of data presented in bar graphs and demonstrated appropriate use of statistical ideas but not in critical ways.

**Table 6:** The distribution of students across the hierarchy by skills and year groups

Skill	Year	Lower group			Upper group		
		L1 N (%)	L2 N (%)	L3 N (%)	L4 N (%)	L5 N (%)	L6 N (%)
Interpreting	Y-9	3 (6%)	18 (37%)	7 (15%)	13 (27%)	5 (11%)	2 (4%)
	Y-12	3 (6%)	7 (15%)	12 (25%)	12 (25%)	11 (23%)	3 (6%)
Communicating	Y-9	0 (0%)	3 (6%)	10 (21%)	31 (65%)	4 (8%)	0 (0%)
	Y-12	0 (0%)	(0%)	0 (0%)	39 (81%)	9 (19%)	0 (0%)
Evaluating	Y-9	2 (4%)	9 (19%)	19 (40%)	16 (33%)	2 (4%)	0 (0%)
	Y-12	0 (0%)	2 (4%)	16 (33%)	23 (48%)	6 (13%)	1 (2%)
Decision-making	Y-9	0 (0%)	6 (12%)	21 (44%)	21 (44%)	0 (0%)	0 (0%)
	Y-12	0 (0%)	1 (2%)	12 (25%)	23 (48%)	10 (21%)	2 (4%)

Table 6 also shows that year 9 students were more likely than year 12 students to demonstrate L2 *interpreting* skill. It also indicates that, when asked to analyze a line graph, the largest proportion of year 9 students (37%) performed at level 2. This suggests they employed informal beliefs. For instance, when interpreting the item with the highest shoe production, they believed that the highest production occurred after lunch, or they associated the highest number of shoes with the largest number on the *y*-axis. Furthermore, less students demonstrated success within the interpreting skill than across the other three skills. The low percentages might be affected by the nature of interpreting task that involved a line graph which was found in other studies as a complex graph representation (Adams & Shrum, 1990; Berg & Phillips, 1994; Patahuddin & Lowrie, 2019).

### The students’ statistical literacy by gender

The first Mann–Whitney *U* test was conducted to examine the effects of gender (females and males) on student’s SL level and the four skills (*interpreting*, *communicating*, *evaluating*, and *decision-making*). The dependent variables were the SL and its four response skills, while the independent variable was the gender (females and males). Table 7 presents the completed Mann–Whitney *U* result for the effects of gender on the students’ SL and skill level.

**Table 7:** A Mann–Whitney *U* test results for SL by gender

Variable	Number of students		Mean rank		*U*	*z*	*P*	In favor of
	Males	Females	Males	Females				
SL	48	48	48.23	48.77	1165	0.109	0.913	None
Interpreting	48	48	53.39	43.61	917.5	−1.761	0.078	None
Communicating	48	48	50.25	46.75	1068	−0.789	0.430	None
Evaluating	48	48	46.85	50.15	1231	0.616	0.538	None
Decision-making	48	48	51.70	45.30	998.5	−1.212	0.226	None

The result of a Mann–Whitney *U* test revealed that SL level was not statistically significantly different between males (mean rank = 48.23) and females (mean rank = 48.77), *U* = 1165, *z* = 0.109, *p* = 0.913, using an exact sampling distribution for *U*. When the four SL skills (*interpreting*, *communicating*, *evaluating*, and *decision-making*) were considered separately, using a Bonferroni adjusted alpha level of 0.013, the results showed no significant statistical differences in *interpreting* skill for males (mean rank = 53.39) and females (mean rank = 43.61), *U* = 917.5, *z* =  − 1.761, *p* = 0.078; in *communicating* skill for males (mean rank = 50.25) and females (mean rank = 46.75), *U* = 1068, *z* =  − 0.789, *p* = 0.430; in *evaluating* skill for males (mean rank = 46.85) and females (mean rank = 50.15), *U* = 1231, *z* = 0.616, *p* = 0.538; and in *decision-making* skill for males (mean rank = 51.70) and females (mean rank = 45.30), *U* = 998.5, *z* =  − 1.212, *p* = 0.226.

### The students’ statistical literacy by grade levels

We then investigated whether there were differences between the SL levels achieved by students in year 9 and year 12. Table 8 presents the completed Mann–Whitney *U* result for the effects of grade level on the students’ SL and skill level.

**Table 8:** A Mann–Whitney *U* test results for SL by grade level

Variable	Number of students			Mean rank	*U*	*Z*	*P*	In favor of
	Y-9	Y-12	Y-9	Y-12				
SL	48	48	40.95	56.05	1514.5	3.041	0.002	Y-12
Interpreting	48	48	43.00	54.00	1416	1.983	0.047	None
Communicating	48	48	40.72	56.28	1525.5	3.508	<0.001	Y-12
Evaluating	48	48	40.31	56.69	1545	3.065	0.002	Y-12
Decision-making	48	48	38.34	58.66	1639.5	3.848	<0.001	Y-12

The result revealed that the SL level was statistically significantly different between year 9 (mean rank = 40.95) and year 12 (mean rank = 56.05), *U* = 1514.5, *z* = 3.041, *p* = 0.002, using an exact sampling distribution for *U*. When the four SL skills (*interpreting*, *communicating*, *evaluating*, and *decision-making*) were considered separately, using a Bonferroni adjusted alpha level of 0.013, the results showed statistically significant differences in *communicating* skill for year 9 (mean rank = 40.72) and year 12 (mean rank = 56.28), *U* = 1525.5, *z* = 3.508, *p* < 0.001; in *evaluating* skill for year 9 (mean rank = 40.31) and year 12 (mean rank = 56.69), *U* = 1545, *z* = 3.065, *p* = 0.002; and in *decision-making* skill for year 9 (mean rank = 38.34) and year 12 (mean rank = 58.66), *U* = 1639.5, *z* = 3.848, *p* < 0.001. There was no significant difference by grade in *interpreting* skill for year 9 (mean rank = 43.00) and year 12 (mean rank = 54.00), *U* = 1416, *z* = 1.983, *p* = 0.047.

## Discussion

This cross-sectional study investigated the level of statistical literacy (SL) of year 9 and year 12 students. Specifically, the investigation examined students’ capacity in the following SL skills: *interpreting*, *communicating*, *evaluating*, and *decision-making*. The results of the analysis revealed that (1) no significant difference was found between males and females in their levels of SL and its four skills; (2) the year 12 students’ SL level was statistically higher than year 9 students; and (3) the analysis revealed differences in favor of year 12 students across the *communicating*, *evaluating*, and *decision-making skills*, but *not interpreting* skill. The discussions on the abovementioned topics are presented below.

*First*, our findings suggest there is no evidence of gender-based disparity in students’ SL levels, which is consistent with findings from large-scale studies for Indonesian high school students. For example, both the PISA 2003 and 2012 results revealed no gender difference in the Indonesian students’ performance on the *uncertainty and data* subscale, which relates directly to the present study, nor the PISA *the change and relationship* and *quantity* subscales (OECD, 2004, 2014). In fact, gender difference was only found in *the space and shape* subscale, in favor of males in the respective PISA studies. This indicates, to a certain extent, in the Indonesian context, both males and females performed similarly in almost all mathematics strands over decades.

*Second*, we anticipated that year 12 students would have better SL skills than year 9 students given the additional instructions they receive in these high school years. This was not the case for the *interpreting* skill. Students, across both grades, found the *interpreting* skill to be more difficult than the other three skills. It may be the case that the unexpected results in terms of this specific skill might be due to the nature of the question rather than the actual skill required to solve the task successfully. Specifically, the task representation (the graphical representation being a line graph) might have been overly complex for students. The task required the interpretation of a line graph of the *Shoes Production*, potentially proving a different layer of complexity to students from both grades. Elsewhere, studies have found that interpreting line graphs can be more difficult for students when compared to the other graphs (Ali & Peebles, 2013; Peebles & Ali, 2015) and even middle school teachers from different contexts with an average of 9 years of teaching experiences misinterpreted line graph representations (Patahuddin & Lowrie, 2019). Furthermore, the capacity to decode graphs is convention-based (Diezmann & Lowrie, 2009; Lowrie & Diezmann, 2007), suggesting that these students have not been exposed to appropriate classroom instruction on how to interpret graphs in these high school years. Consequently, future studies might seek to determine the students’ capacity to understand the graph conventions as well as the statistical content when investigating the students’ SL.

In general, having more year 12 than year 9 students in L4 (*consistent non-critical*) and L5 (*critical*) bands confirmed the pre-assumption that higher-grade students have better SL. Such progress in the students’ SL by grades is consistent with the previous studies (e.g., Aoyama & Stephens, 2003; Callingham & Watson, 2017; Yolcu, 2014). Moreover, statistical analysis confirmed that the SL level of year 12 students was significantly higher than year 9 students with a medium effect size of 0.310 (Tomczak & Tomczak, 2014). Given the year 9 students were coming from a relatively low base of performance (evidenced by 42% of year 9 students in the lower group), however, this medium effect size growth by year 12 is surprising. The students’ SL is still a concern due to the fact that the majority of years 9 and 12 students’ performance banding was clustered at L4, suggesting students typically interpret the quantitative information without questioning. Thus, on average, students in both grades only achieved the minimum level required for adults’ SL according to Klein et al. (2016).

Based on those results, the future participation of the year 12 students in an information-driven society post school is of concern. Such concern includes not only the modal level achieved by the students (which is approaching L4), but also the absence of students in the critical mathematical level (L6). The absence of Indonesian year 9 students in the highest level might have been predicted given their underperformance in PISA *uncertainty and data* over 2 decades. However, the year 12 students’ absence in the L6 (*critical mathematical*) is somewhat problematic as this would be the benchmark for their participation in society. Moreover, they will unlikely get more formal and professional statistics instruction unless they continue to study at university with statistics as their major. Their lack of critical response could become an issue if it continues and thus could turn them into not-sufficiently informed citizens.

## Conclusion and implications

The results of this study offer useful contributions to both the field of assessment and learning associated with SL, particularly for the Indonesian high school students. From an assessment perspective, the framework can be used to monitor and measure students’ SL knowledge from the data consumers’ perspective. In fact, the framework offers the flexibility to also provide a more detailed analysis in the students’ SL levels, by skills. From a learning perspective, the assessment framework can be used to target individuals learning progress (formatively) and align instruction to the specific skill (e.g., *evaluating* statistical claim).

The absence of students in the critical mathematical level highlights further pedagogical implications for the study. The statistics instruction in Indonesian high schools should offer opportunities for students to be exposed to critical statistical thinking. We recommend that teachers incorporate statistical information from different contexts and with various representations (particularly line graph) in their statistics lessons via online or printed media. The teachers could facilitate the students to critically respond (*interpret*, *communicate*, *evaluate*, and *make decision*) to such information. This would be a great experience for students to practice their critical thinking when encountering statistical information in real life. The way students perform critical response when *interpreting,* c*ommunicating*, *evaluating*, and *making decision* could also be observed.

Finally, three limitations are evident in this study. First, data were sourced from two cities in one province in Indonesia. Although the results can still be generalized to students whose context is similar to this study, nevertheless, a larger sample size would provide a more reliable measure of the Indonesian students’ SL. Second, the quality of the items selected for analyses. Those items were sourced from national and international tests that they may not always be of high quality, but they do represent current examples of SL assessment. Furthermore, the results of the present study were based on eight items, and each SL skill was measured by two items. Each skill included only one out of three data representations (i.e., table, bar graph, or line graph). Thus, further study is needed to cover more items using different types of representation to assess each skill.

Third, the current investigation presented SL items that contain graphic element. We acknowledge that SL items do not necessarily require a graphic; consequently, our analysis is limited to data that contain a graphicacy component. Elsewhere, Gal and Geiger (2022) noted that new demand on SL goes beyond those elements contained in the eight questions presented in this study. Gal and Geiger (2022) identified nine separate categories of information that is typically included in items that require the coding of SL. In their analysis, not all SL items required the interpretation of a graphic; however, our findings are restricted to the students’ capacity to interpret graphical information. As Friel et al. (1997) mentioned, graph tasks require the decoding of data that can involve reading the data, reading between the data, and reading beyond the data. Future studies should include non-graphic items in order to ascertain SL beyond those tasks bounded by graphics.

## Appendix A. The descriptors of the six hierarchical level

**Table:**

Level	General descriptors	Item-related descriptor for *The 100-m race* item
*Critical mathematical*	**Text & Context:** Student shows critical and questioning engagement with both familiar and unfamiliar contexts **Representation**: Student shows ability to summarize the association of the variables shown in the graph/table and relate it to the context **Statistical-mathematical**: Student performs sophisticated or critical statistical and mathematical skills, associated with mathematical concepts such as central tendency and dispersion measures	**Text & Context**: Student understands that choosing the best runner cannot be done in one way, it needs to consider and compare several appropriate ways; possibly interprets the data out of the provided context critically **Representation**: Student provides a critical interpretation of the times presented in the table for each runner; appropriately identifies trends and measure of data center **Statistical-mathematical**: Student is able to select the best runners by considering various statistical ideas such as mean, trend, mode, and variation; combining mean and trends; and performing accurate and sophisticated calculations
*Critical*	**Text & Context:** Student shows critical engagement with familiar context and non-critical with unfamiliar contexts **Representation**: Student demonstrates awareness of relevant features of graph/table and awareness of the integration of more than one relevant aspect of data **Statistical-mathematical**: Student shows qualitative interpretation and sophisticated use of mathematics or statistical concepts	**Text & Context**: Student understands the context as the previous level but can decide how to choose one runner critically **Representation**: Student focuses on the times of all participants (can relate the information in the table to the context to find the mean of time of the three runners); can compare the time from race to race for all three runners **Statistical-mathematical**: Student is able to choose one of two runners who have the same mean with further critical justification (such as the fastest record, won the most races, etc.); compares trends and interprets them from the three runners; uses of basic number sense in calculating the mean
*Consistent non-critical*	**Text & Context:** Student shows appropriate engagement with the context but often in a non-critical manner **Representation**: Student makes sense the data presented in graph/table with partial recognition of the context, focuses on a single relevant aspect, or compares within a data table or graph **Statistical-mathematical**: Student reasonably shows the application of statistical and mathematical concepts and includes those associated with graph characteristics	**Text & Context**: Student understands that the winner of the race is the runner who has (the shortest) time (in each race/total time); considers the time taken by a runner from race to race; considers the runner who won most often (or running with the shortest time) **Representation**: Student focuses on each race (finds the smallest time in each race and or after those times are added up); student compares the decreasing time from race to race of a runner **Statistical-mathematical**: Student only uses one of the mode/trend/total time/mean; chooses two runners who have the same mean; chooses the runner who has the lowest mean due to miscalculation
*Inconsistent*	**Text & Context:** Student shows selective or inconsistent engagement with the context (dependent to some extent on the format of the items) **Representation**: Student tends to interpret the graphical/tabular details rather than the context in graph/table and relate it to irrelevant contextual issues **Statistical-mathematical:** Student makes conclusions but may not be accompanied by suitable statistical or mathematical justifications	**Text & Context**: Student begins to understand (but still involves informal understanding) the context of the running competition; understand the context of selecting one out of three runners but do not understand the context of the time required by runners such as choosing the runner who wins the most (with the largest number) **Representation**: Student focuses on finding the winner (= the longest time) in each competition/after adding up/calculating the mean; begins to understand the meaning of the numbers on the table for each runner but it is not consistent **Statistical-mathematical**: Student performs errors in selecting one runner such as using the mode to select the best runner (with the greatest time); performs procedural calculations (allowing a slight error in the calculation, but still chooses the one with the greatest total time/biggest mean); Even though the understanding is correct, there is a fatal miscalculation so that the choice is wrong
*Informal*	**Text & Context:** Student shows engagement with the context but colloquial or informal (reflecting intuitive or non-statistical believe) and reflects irrelevant aspects of the context **Representation**: Student is able to observe the value presented in the graph/table **Statistical-mathematical:** Student performs basic one-step table and graph calculation (such as addition and subtraction) based on the values observed, yet sometimes with imaginative story	**Text & Context**: Student relates the context of choosing runners with other contexts, such as choosing the best, highest, greatest value, etc.; connects the data in the table with the strength of each runner **Representation**: Student focuses on finding the largest numbers from the table as a whole; interprets based on everyday/other informal understanding of the data in the table; interprets the increasing time in a positive way and the decreasing time in a negative way **Statistical-mathematical**: Student performs addition of certain numbers and does not relate to the question; makes a lot of miscalculations; selects a runner based on an informal contextual understanding and table reading
*Idiosyncratic*	**Text & Context:** Student shows non-existent and personal engagement with the context **Representation**: Student shows personal belief and experience underlying their basic graph and table reading (e.g., reading cell values) **Statistical-mathematical:** Student guesses the answer, makes one-to-one counting, picks a random value, selects the largest number or other unreasonable steps	**Text & Context**: Student skips the information in the text above the table / fails to relate the information in the text to the numbers in the table; involves personal (non-statistical) views in interpreting the context of the 100-m race **Representation**: Student only mentions/re-writes numbers in table without knowing their meaning; does not know the meaning of the numbers 1–7 (= the 1–7) and/or the numbers in the table (= the time required for each student); provides no results from the table reading **Statistical-mathematical**: Student selects one runner with no reason; does not perform any statistical and mathematical knowledge
